# Albuminuria and Dipstick Proteinuria for Predicting Mortality in Heart Failure: A Systematic Review and Meta-Analysis

**DOI:** 10.3389/fcvm.2021.665831

**Published:** 2021-05-13

**Authors:** Wei Liang, Qian Liu, Qiong-ying Wang, Heng Yu, Jing Yu

**Affiliations:** ^1^Department of Cardiology, Lanzhou University Second Hospital, Lanzhou, China; ^2^Research Center, Gansu Provincial Maternal and Child Care Hospital, Lanzhou, China

**Keywords:** proteinuria, albumin, mortality, cardiac failure, meta-analysis

## Abstract

**Background:** Research suggest that albuminuria is not only an independent risk factor for the development of heart failure but may also act as a biomarker for predicting adverse outcomes. To date, no study has synthesized evidence on its role as a prognostic indicator. Thus, the current study aimed to quantitatively assess the prognostic utility of albuminuria as well as dipstick proteinuria in predicting mortality in heart failure patients.

**Methods:** PubMed, Embase, ScienceDirect, CENTRAL, and Google Scholar databases were searched up to October 10, 2020. All studies reporting multivariable-adjusted hazard ratios (HR) for albuminuria or dipstick proteinuria for mortality and/or hospitalization in heart failure patients were included.

**Results:** Eleven studies were included. Seven assessed albuminuria and five assessed dipstick proteinuria. Our analysis revealed a statistically significant increased risk of all-cause mortality with microalbuminuria (HR: 1.54; 95% CI, 1.23–1.93; *I*^2^ = 79%; *p* = 0.0002) and macroalbuminuria (HR: 1.76; 95% CI, 1.21–2.56; *I*^2^ = 88%; *p* = 0.003) in heart failure patients. The risk of all-cause mortality and hospitalization was also significantly increased with macroalbuminuria. Microalbuminuria was associated with significantly increased cardiovascular mortality and combined cardiovascular mortality and hospitalization. Positive dipstick test for proteinuria was significantly associated with mortality in heart failure (HR: 1.54; 95% CI, 1.28–1.84; *I*^2^ = 67%; *p* < 0.00001).

**Conclusion:** Both microalbuminuria and macroalbuminuria are predictors of mortality in patients with heart failure. Dipstick proteinuria may be used as a rapid screening test to predict mortality in these patients.

## Introduction

Heart failure has been identified as a major cardiovascular disease leading to a global public health problem. According to recent estimates, around 64.3 million individuals are affected by heart failure worldwide, with a ratio of 8.5 patients per 1,000 individuals (1). Such high numbers account for 9.9 million years lost due to disability from the disease (1). Heart failure has been associated with significant morbidity, mortality as well as high healthcare expenditure. Despite immense research and developments in the management of the disease, hospitalization for heart failure, incidence of readmissions, and death rates remain high (2). To prevent mortality and hospitalizations due to heart failure, it is necessary to extensively characterize the predictors of such adverse events, amalgamating it with the impact of other comorbidities present in such patients.

Traditional risk factors like diabetes, hypertension, smoking, and hyperlipidemia are well-recognized for predicting mortality with cardiovascular diseases (3, 4). Studies have also reported that albuminuria not only is an independent risk factor for the development of cardiovascular disease but also acts as a biomarker for predicting mortality in such patients (5–7). Specifically, in heart failure patients, the presence of albumin in the urine can represent several pathophysiological conditions like systemic inflammation as well as endothelial and microvascular dysfunction, which may have a role in the prognosis of the disease (8). Furthermore, it may represent kidney injury due to heart failure itself or other comorbidities like diabetes and chronic kidney disease (9). Studies indicate that albuminuria can be a potential target to improve clinical outcomes in heart failure patients (10).

In the past two decades, a few studies have specifically focused on the role of albuminuria in predicting adverse outcomes in heart failure patients (11–13). Owing to the diverse population of the included studies along with adjustment of different confounding factors, there is a need for a pooled analysis to determine the exact value of albuminuria as a biomarker for predicting mortality and hospitalization in heart failure patients. Furthermore, dipstick proteinuria has been suggested as an alternative to the more cumbersome testing of 24-h urinary albumin-to-creatinine ratio for detecting albuminuria (7). Thus, in the current study, we aimed to quantitatively assess the prognostic utility of albuminuria as well as dipstick proteinuria in predicting mortality and hospitalization in heart failure patients.

## Materials and Methods

### Inclusion Criteria

The review is conducted as per the guidelines of the Preferred Reporting Items for Systematic Reviews and Meta-analyses (PRISMA) statement (14). We included studies with the following inclusion criteria: (1) conducted on patients with heart failure, (2) comparing outcomes of patients with and without albuminuria or dipstick proteinuria, (3) reporting mortality data, and (4) data reported as adjusted hazard ratios (HR) with 95% confidence intervals (CI). No restriction was placed on the study design, sample size, or date of publication. The following were the exclusion criteria for the review: (1) studies on a mixed population of cardiovascular diseases, (2) studies not reporting relevant outcomes, (3) studies reporting unadjusted HR, (4) review articles, case reports, and unpublished studies, and (5) non-English language studies.

### Search Strategy

An electronic search was conducted by two reviewers, independent of each other, for the following databases: PubMed, Embase, ScienceDirect, CENTRAL, and Google Scholar. The time limit was from the inception of the databases to October 10, 2020. The terms used for the literature search included the following: “heart failure,” “cardiac failure,” “albuminuria,” “proteinuria,” “albumin,” “urinary,” “death,” and “mortality.” The search terms were used in different combinations to find relevant articles. After the deduplication of articles, the search records were analyzed separately by their titles and abstracts by the two reviewers. Articles matching the inclusion criteria were identified, and the full texts of these were extracted. Individual studies were then assessed for final inclusion in the study. Any disagreements were resolved by discussion. After completion of the search and identification of included studies, the bibliography of included articles was manually searched for any other potential article.

### Data Extraction and Quality of Included Studies

The following data were extracted from the included studies: names of first authors, publication year, study type and location, use of albuminuria or dipstick proteinuria, definition of albuminuria, sample size, demographic details of the sample, outcomes reported, adjusted variables for outcomes, and follow-up time. The primary outcome of interest was mortality (both all-cause and cardiovascular mortality). The secondary outcome of interest was the risk of hospitalization for heart failure.

The risk of a bias was assessed using the Newcastle–Ottawa Scale (15). Two reviewers independently assessed each study. Studies with more than seven points were judged to be of high quality. Any disagreements were resolved by discussion.

### Statistical Analysis

“Review Manager” [RevMan, version 5.3; Nordic Cochrane Centre (Cochrane Collaboration), Copenhagen, Denmark; 2014] was used for the meta-analysis. We extracted data on adjusted HR for mortality and hospitalization for heart failure as reported by the included studies and combined them using inverse variance-weighted averages of logarithmic HRs. Meta-analysis was conducted only if at least two studies reported the same outcome. Sub-group analysis was conducted for microalbuminuria and macroalbuminuria. Studies reporting dipstick proteinuria were pooled separately. In studies reporting data for different sub-groups for evaluating the prognostic significance of albuminuria or dipstick proteinuria, we combined data of the sub-groups into one singular group using the meta-analysis software itself. The pooled data of all sub-groups from a given study was then used for the meta-analysis. We assessed heterogeneity using the *I*^2^ statistic. *I*^2^-values of 25–50% represented low, values of 50–75% medium, and values more than 75% represented substantial heterogeneity. Due to the different study designs, the difference in factors adjusted, and the varied study populations of the included studies, we chose to use the random-effects model for the meta-analysis. As <10 studies were included in the meta-analysis, funnel plots were not used to assess publication bias. We also conducted a sensitivity analysis for the outcome of all-cause mortality to assess if any study had an undue influence on the results. Each study in the analysis was sequentially excluded to recalculate the effect size in the meta-analysis software itself.

## Results

### Characteristics of the Included Studies

**Figure 1:**
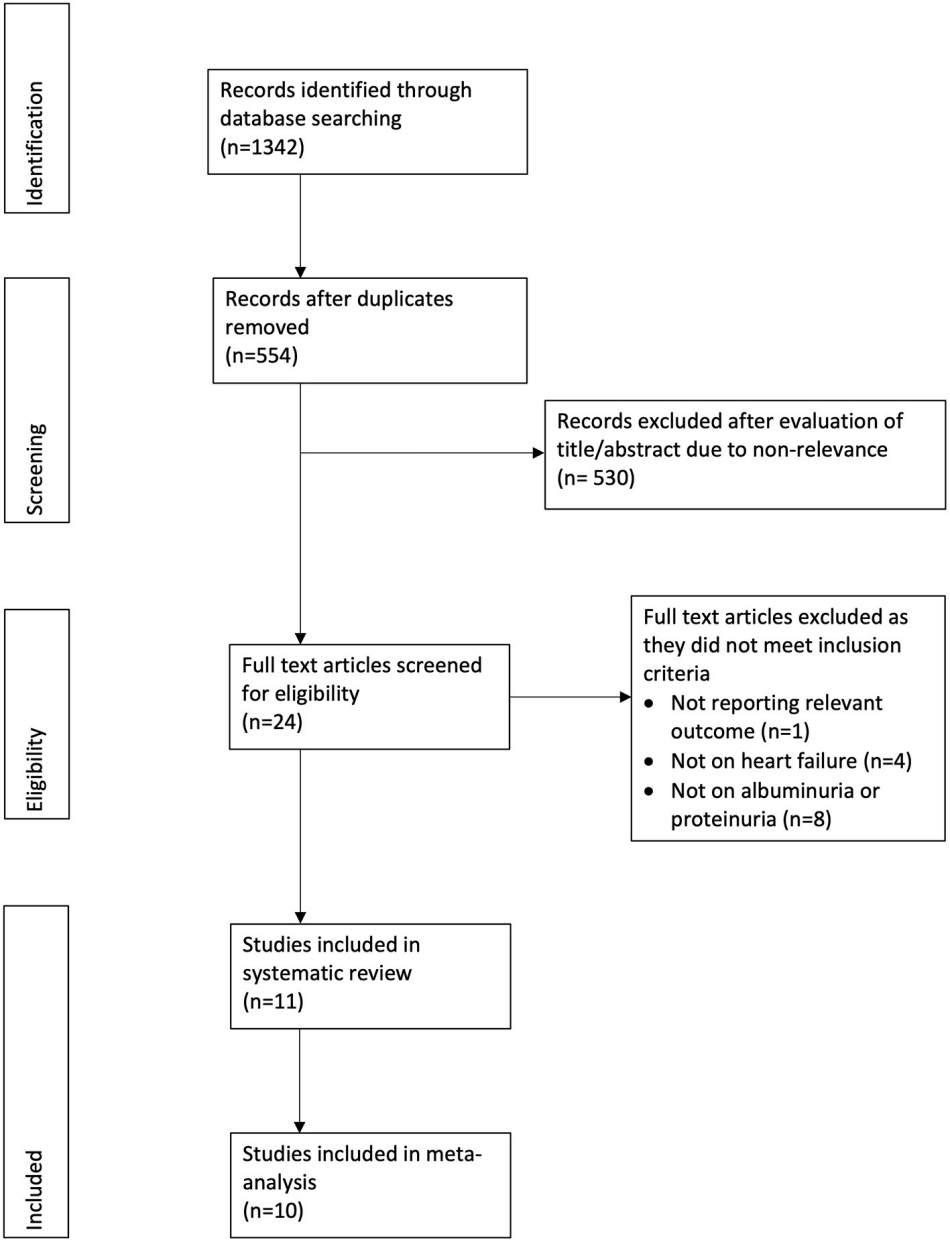
Study flow chart.

The PRISMA flow chart of the review is presented in Figure 1. A total of 12 studies were included in the review. Seven studies (11–13, 16–19) evaluated albuminuria using 24-h urinary protein excretion in a central laboratory, while five studies (20–24) assessed proteinuria using the dipstick test. We separated these studies for this analysis. The details of seven studies evaluating albuminuria are presented in Table 1. Four studies were secondary analysis of randomized controlled trials (RCTs) from the baseline population, while two were retrospective studies and one was a prospective study. The sample size in these studies ranged from 712 to 4,668 patients. The mean ejection fraction was ≤ 50% in three studies. Similar definitions of microalbuminuria and macroalbuminuria were used in the included studies except for that of Jackson et al. (12). The follow-up duration and variables adjusted for the multivariate analysis differed across studies. None of the included studies were of low quality.

**Table 1:** Characteristics of included studies on albuminuria.

**References**	**Study type**	**Study location**	**Sample size**	**Mean age**	**Male gender (%)**	**Mean** **LVEF (%)**	**NYHA III/IV (%)**	**Definition of albuminuria**	**Outcome of interest reported**	**Follow-up**	**Covariates adjusted**	**NOS score**
Shuvy et al. (19)	Retrospective	Israel	4,668	76 (67–84)	56	NR	40	Microalbuminuria: 30–300 mg/g Macroalbuminuria: >300 mg/g	Mortality, hospitalization for heart failure	720 days	Heart disease, DM, hypertension, atrial fibrillation, log-transformed serum urea levels, square root-transformed eGFR, hemoglobin, serum sodium, drug treatment with angiotensin-converting enzyme inhibitor/angiotensin receptor blocker, beta blocker, furosemide, spironolactone, and aspirin	7
Selvaraj et al. (18)	RCT	Multiple countries	1,175	72 ± 9.6	52.2	58	66.2	Microalbuminuria: 30–300 mg/g Macroalbuminuria: >300 mg/g	Mortality, hospitalization for heart failure	3.5 years	NYHA class, DM, serum creatinine, heart rate, age, sex, race, smoking status, atrial fibrillation, peripheral artery disease, ejection fraction, systolic blood pressure, assignment to spironolactone vs. placebo	8
Liu et al. (17)	Retrospective	China	1,474	65.5 ± 6.5	70.8	NR	NR	Microalbuminuria: 30–300 mg/g Macroalbuminuria: >300 mg/g	Mortality, hospitalization for heart failure	56 months	Age, sex, heart rates, systolic blood pressure, diastolic blood pressure, DM, prior myocardial infarction, stroke/transient ischemic attack, eGFR, statin, diuretics, angiotensin-converting enzyme inhibitor or angiotensin II receptor antagonist, snoring, alcohol consumption, smoking, physical activity	8
Katz et al. (16)	RCT	Multiple countries	144	66 ± 11	38	62	58	Microalbuminuria: 30–300 mg/g Macroalbuminuria: >300 mg/g	Mortality and hospitalization for heart failure	12.1 months	Age, sex, African-American race, DM, kidney disease, coronary artery disease, anemia, and various markers of cardiac disease severity, including brain natriuretic peptide, left ventricular mass index, E/e' ratio, and NYHA functional class	7
Niizeki et al. (13)	Prospective	Japan	712	73 ± 14	63	46	52	Microalbuminuria: 30–300 mg/g Macroalbuminuria: >300 mg/g	Mortality and hospitalization for heart failure	1,500 days	Age, systolic blood pressure, cardiothoracic ratio, sodium, hemoglobin, uric acid, eGFR, cystatine C, phosphorus, and brain natriuretic peptide	
Masson et al. (11)	RCT	Italy	2,131	67 ± 11	78.9	33	30.1	Microalbuminuria: 30–300 mg/g Macroalbuminuria: >300 mg/g	Mortality	3 years	Age, sex, NYHA class, LVEF, etiology, systolic and diastolic blood pressures, heart rate, prescription of angiotensin-converting enzyme inhibitors, B-blockers, or diuretics, atrial fibrillation, chronic obstructive pulmonary disease or DM, serum concentrations of potassium, creatinine, and triglycerides	8
Jackson et al. (12)	RCT	Multiple countries	2,310	68.3 ± 10.2	65.2	40	62.5	Microalbuminuria: men: 2.5–25 mg/mmol Women: 3.5–25 mg/mmol Macroalbuminuria: >25 mg/mmol	Mortality, hospitalization for heart failure	37.7 months	Randomly assigned treatment (candesartan vs. placebo), sex, NYHA class, smoking habit, age, LVEF, body mass index, systolic blood pressure, diastolic blood pressure, heart rate, history (admission for heart failure, myocardial infarction, angina pectoris, stroke, hypertension, DM, atrial fibrillation, cancer, coronary artery bypass surgery, percutaneous coronary revascularization, implanted cardioverter defibrillator, or pacemaker), and baseline treatment (diuretic, digitalis, β blocker, angiotensin-converting enzyme inhibitor, calcium channel blocker, other vasodilators, antiarrhythmic drug, lipid-lowering drug, anticoagulant, aspirin, and other antiplatelets)	8

*NOS, Newcastle–Ottawa Scale; NR, not reported; NYHA, New York Heart Association; LVEF, left ventricular ejection fraction; RCT, randomized controlled trial; DM, diabetes mellitus; eGFR, estimated glomerular filtration rate*.

Table 2 presents the details of studies using the dipstick examination method for assessing proteinuria. Of these five studies, three were secondary analyses of RCTs, while two were retrospective studies. The sample size ranged from 1,056 to 24,331 patients in the included studies. One study presented data on in-hospital mortality, while others documented long-term mortality. None of the included studies were of low quality.

**Table 2:** Characteristics of included studies on dipstick proteinuria.

**References**	**Study type**	**Study location**	**Sample size**	**Mean age**	**Male gender (%)**	**Mean LVEF (%)**	**NYHA III/IV (%)**	**Outcome reported**	**Follow-up**	**Covariates adjusted**	**NOS score**
Chen et al. (23)	Retrospective	China	1,056	72.5	56.5	39.6	NA	In-hospital mortality	30 days	Gender, smoking status, hypertension, stroke, chronic obstructive pulmonary diseases, C-reactive protein, LVEF, and renin–angiotensin system inhibitor therapy at admission, NT-proBNP, diabetes, renin-angiotensin system inhibitors, albumin	7
Brisco et al. (24)	RCT	Multiple countries	6,439	59.4 ± 10.2	85.8	27	NR	Mortality	2.8 years	Age, race, sex, hypertension, diabetes, cerebrovascular disease, ischemic HF etiology, ejection fraction, NYHA class, heart rate, systolic and diastolic blood pressure, beta blocker use, digoxin use, loop and potassium-sparing diuretic use, hematocrit, serum sodium, baseline eGFR, and study drug	8
Smith et al. (20)	Retrospective	USA	24,331	74.1 ± 12	52.4	NR	NR	Mortality, hospitalization for heart failure	22.1 months	Age, sex, prevalent heart failure, acute myocardial infarction, unstable angina, percutaneous coronary intervention, coronary artery bypass surgery, ischemic stroke or transient ischemic attack, other thromboembolic event, atrial fibrillation or flutter, mitral or aortic valve disease, peripheral arterial disease, rheumatic heart disease, implantable cardioverter defibrillator, pacemaker, dyslipidemia, diabetes mellitus, hospitalized bleeds, diagnosed dementia, chronic liver disease, chronic lung disease, mechanical fall, systemic cancer, hemoglobin, systolic blood pressure, high-density lipoprotein cholesterol, low-density lipoprotein cholesterol, year of study entry, and sites	7
Miura et al. (22)	RCT	Japan	2,465	69.6 ± 11.6	68.2	65	8.6	Mortality	2.5 years	Age, sex, and clinical status (NYHA class, systolic blood pressure, heart rate, body mass index, LVEF), serum sodium, serum potassium, history of malignant tumor, admission for heart failure, and comorbidities (diabetes, hyperuricemia, anemia, coronary artery disease, cerebrovascular disease, atrial fibrillation), five urine dipstick test brands, and treatment drugs	8
Anand et al. (21)	RCT	Multiple countries	5,010	62.7 ± 11	80	26.8	38.1	Mortality	23 months	Male gender, age ≥65 years, race, ischemic heart disease, hemoglobin, atrial fibrillation, diabetes mellitus, systolic blood pressure, pulse rate, peripheral edema, NYHA functional class, LVEF, plasma sodium, plasma potassium, plasma albumin, brain natriuretic peptide, neutrophil count, lymphocyte count, norepinephrine, aldosterone, plasma renin activity; use of digoxin, an angiotensin-converting enzyme inhibitor, a B-blocker, aspirin, spironolactone, or a diuretic; and randomly assigned treatment (valsartan or placebo)	7

*NOS, Newcastle–Ottawa Scale; NR, not reported; NT-proBNP, N-terminal pro-brain natriuretic peptide; NYHA, New York Heart Association; LVEF, left ventricular ejection fraction; RCT, randomized controlled trial*.

### Meta-Analysis

**Figure 2:**
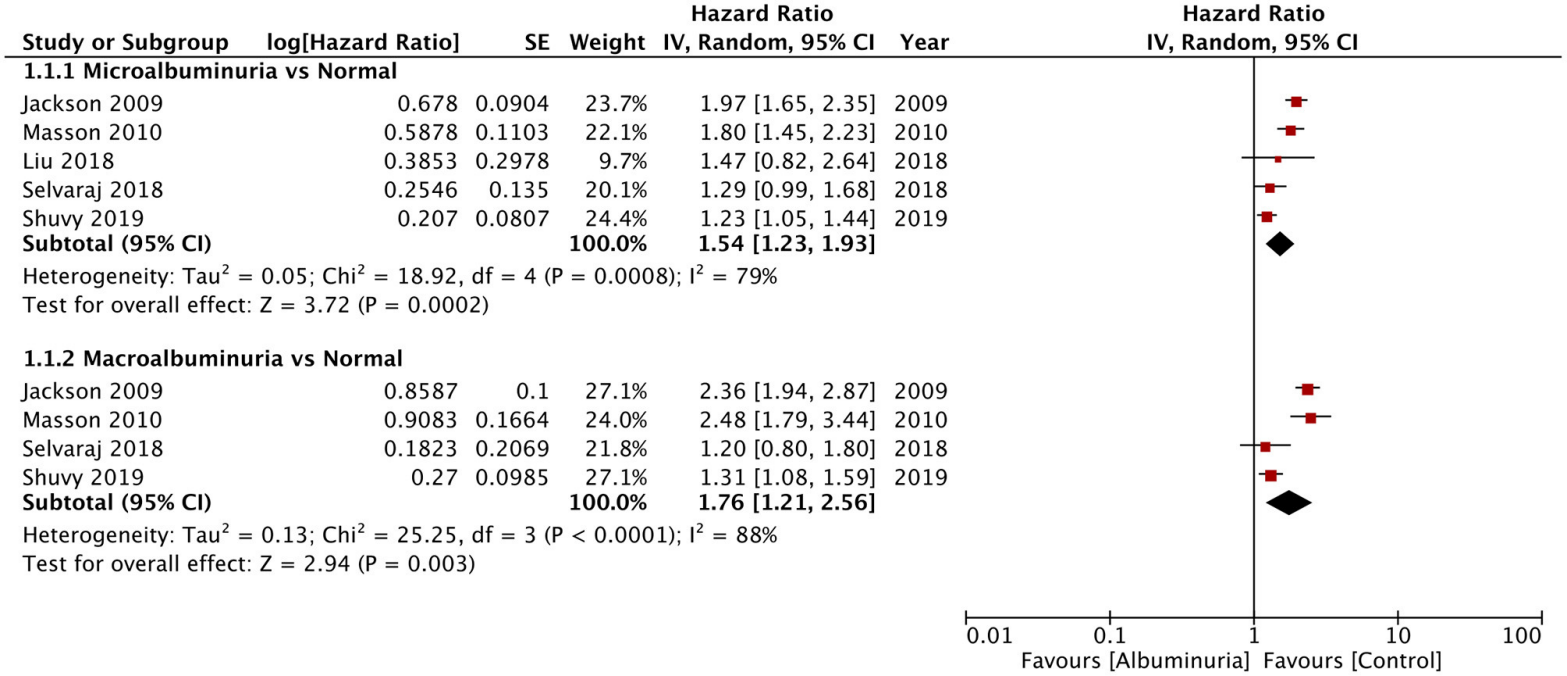
Forest plot for all-cause mortality with albuminuria in heart failure patients.

**Figure 3:**
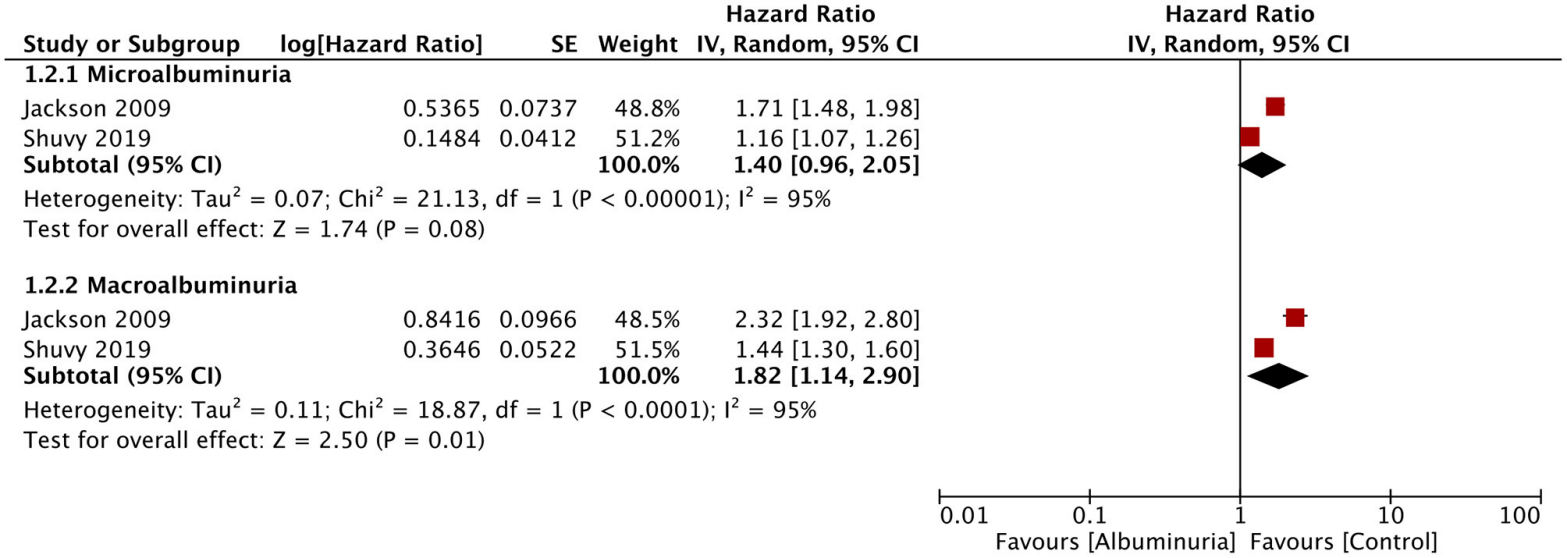
Forest plot for all-cause mortality and hospitalization for heart failure with albuminuria in heart failure patients.

**Figure 4:**
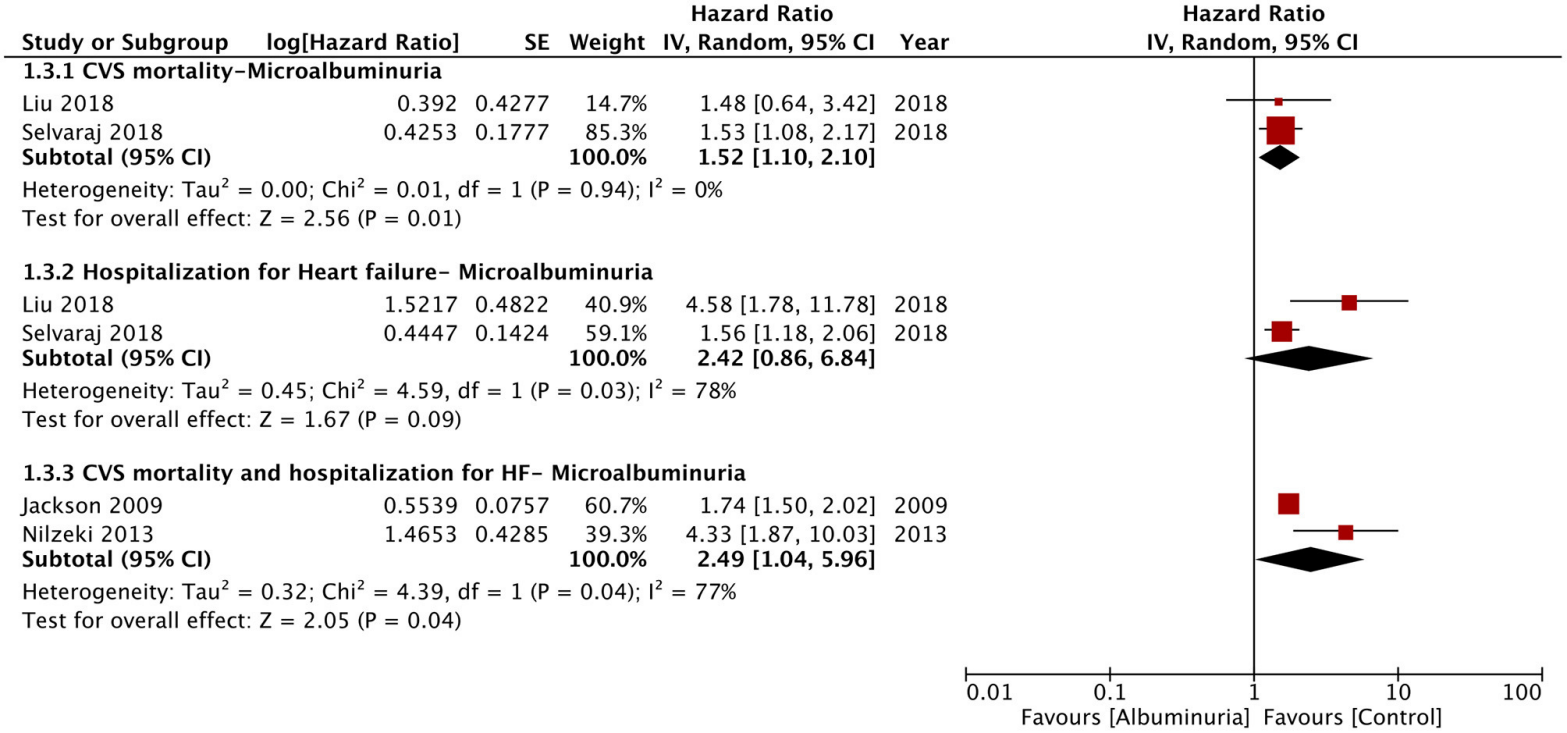
Forest plot for cardiovascular mortality and hospitalization for heart failure associated with microalbuminuria in heart failure patients.

Of the seven studies reporting 24-h urine albuminuria measurements, one study analyzed the relationship of albuminuria and mortality by treating albuminuria as a continuous variable, unlike other studies that grouped the patients into microalbuminuria or macroalbuminuria. Hence, this study was excluded from the meta-analysis. On descriptive analysis, the study of Katz et al. (16) reported a statistically significant increase in mortality per doubling of urinary albumin (HR: 1.15; 95% CI, 1.04–1.28) in a multivariable-adjusted model. On the pooling of data of all-cause mortality from five studies, our analysis revealed a statistically significant increased risk of mortality with microalbuminuria (HR: 1.54; 95% CI, 1.23–1.93; *I*^2^ = 79%; *p* = 0.0002) and macroalbuminuria (HR: 1.76; 95% CI, 1.21–2.56; *I*^2^ = 88%; *p* = 0.003) (Figure 2). On sensitivity analysis, there was no change in the significance of the results on sequential exclusion of each study. Two studies reported HR for all-cause mortality and hospitalization. Pooled analysis revealed a significantly increased risk of this composite outcome for macroalbuminuria (HR: 1.82; 95% CI, 1.14–2.90; *I*^2^ = 95%; *p* = 0.01) but not microalbuminuria (HR: 1.40; 95% CI, 0.96–2.05; *I*^2^ = 95%; *p* = 0.08) (Figure 3). Our analysis also revealed a significantly increased risk of cardiovascular mortality (HR: 1.52; 95% CI, 1.10–2.10; *I*^2^ = 0%; *p* = 0.01) and combined cardiovascular mortality and hospitalization for heart failure (HR: 2.49; 95% CI, 1.04–5.96; *I*^2^ = 77%; *p* = 0.04) in patients with microalbuminuria (Figure 4). Analysis of data from two studies, however, did not reveal statistically significant results for the singular outcome of hospitalization for heart failure in patients with microalbuminuria (HR: 2.42; 95% CI, 0.86–6.84; *I*^2^ = 78%; *p* = 0.09) (Figure 4).

**Figure 5:**
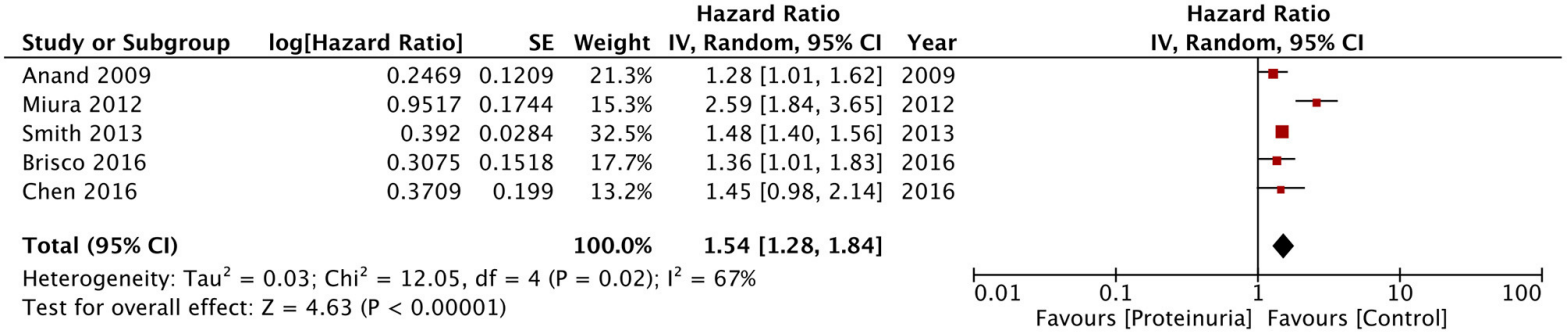
Forest plot for all-cause mortality with dipstick proteinuria in heart failure patients.

In the second part of the meta-analysis, we pooled the data of the five studies using dipstick proteinuria as a prognostic indicator for mortality in heart failure patients. The pooled analysis indicated a statistically significant increased risk of mortality in heart failure patients with a positive dipstick test for proteinuria (HR: 1.54; 95% CI, 1.28–1.84; *I*^2^ = 67%; *p* < 0.00001) (Figure 5). On sensitivity analysis, there was no change in the significance of the results on sequential exclusion of each study. For this analysis, Miura et al. (22) reported separate data for patients with and without low estimated glomerular filtration rate (eGFR) (defined as eGFR of < 60 ml/min/1.73 m^2^) but with positive dipstick test. Their results were statistically significant for both groups with HR of 2.71 (95% CI, 1.72–4.27) for patients with low eGFR and HR of 2.44 (95% CI, 1.47–4.05) for patients with normal eGFR. Data for both groups were combined for the meta-analysis. On the other hand, Smith et al. (20) reported separate data based on preservation of systolic function (preserved or reduced) and the amount of proteinuria traced on the dipstick test (+1, +2, and +3). Data for these sub-groups was combined for the analysis. For the study of Brisco et al. (24), data of high blood urea nitrogen/creatinine and low blood urea nitrogen/creatinine groups was combined for the meta-analysis. Owing to the difference in sub-groups of the individual studies, sub-group analysis for dipstick proteinuria could not be conducted in our review.

## Discussion

Our study, which is the first systematic review and meta-analysis in the literature assessing the impact of albuminuria and dipstick proteinuria on the prognosis of heart failure, presents the following important findings: (1) presence of microalbuminuria in heart failure patients increases the risk of all-cause mortality by around 54%, while macroalbuminuria increases the risk by 76%, (2) limited studies indicate that the risk for the combined outcome of all-cause mortality and hospitalization for heart failure is increased by 82% in the presence of macroalbuminuria, (3) microalbuminuria increases the risk of cardiovascular mortality by around 52% and combined cardiovascular mortality and hospitalization by 149%, and (4) positive dipstick proteinuria in heart failure patients is associated with 54% increased risk of all-cause mortality.

Albuminuria or proteinuria is a recognized biomarker for impaired glomerular filtration and kidney disease. Studies in literature have reported a significant prevalence of albuminuria in heart failure patients ranging from 41% to as high as 54% (12, 19). Such high prevalence has also been reported in studies analyzing patients already on medications such as angiotensin-converting enzyme inhibitors or angiotensin receptor antagonists which are known to reduce the prevalence of albuminuria (18). The high prevalence of albuminuria in heart failure can be attributed to the concomitant presence of other illnesses like diabetes mellitus, hypertension, dyslipidemia, and chronic kidney disease (5). However, studies have shown that the presence of albuminuria is not only restricted to the sub-population with comorbidities but is also prevalent in patients without them. In the real-world data of Shuvy et al. (19), 22% of patients without comorbidities had the presence of albuminuria. In such cases, the pathological role of heart failure itself has been implicated. The hemodynamic state and raised central venous pressure in heart failure patients can cause vascular congestion in the kidneys and reduced renal blood flow. Glomerular hypertension along with tubulointerstitial hypoxia can reduce the number of nephrons, leading to hyperfiltration and albuminuria (5). The decline in renal function is then further complimented by systemic inflammation, oxidative stress, and endothelial dysfunction due to heart failure and other comorbidities like diabetes which aggravate the filtration of albumin through the glomerular basement membrane and accelerate cardiac damage (5, 13, 25). The association between heart failure and albuminuria seems plausible as increased severity of heart failure has been shown to increase the risk of albuminuria (19).

The association between albuminuria and increased cardiovascular mortality is established in diseases like diabetes and hypertension. Toyama et al. (26), in a systematic review and meta-analysis of 31 studies, have reported an increased risk of cardiovascular mortality with microalbuminuria [risk ratio (RR), 1.76; 95% CI, 1.38–2.25] and macroalbuminuria (RR, 2.96; 95% CI, 2.44–3.60) as compared to normoalbuminuria in diabetics. Guidelines for the management of hypertension recommend screening as well as targeted management of albuminuria in addition to controlling blood pressure for better clinical outcomes (27). Despite evidence suggesting the deleterious effects of albuminuria independent of kidney function in patients with other cardiovascular diseases (28, 29), there has been limited research on the prognostic significance of this biomarker specifically in heart failure patients. In this context, our review presents important pooled evidence suggesting a significant role of albuminuria in the worsening prognosis of heart failure. The increased risk of mortality was seen with both microalbuminuria and macroalbuminuria, indicating that even mild albuminuria has a prognostic significance in these patients. Evidence was obtained from a pooled analysis of multivariable-adjusted hazard ratios with different studies taking into account multiple confounding factors to arrive at the statistical figure. This lends credibility to the meta-analysis conducted in our review. Furthermore, the finding was stable on sensitivity analysis, with no study exerting an undue influence on the results. It can also be noted from the forest plot of all-cause mortality that the effect size was larger in earlier as compared to more recent studies. While the exact reason for this is difficult to decipher owing to the limited number of studies in our review, the improvement in the management of heart failure as well as associated comorbidities over the period of time may have contributed to this difference.

Our findings have important clinical implications as targeting albuminuria in heart failure patients can be an important therapeutic approach. Studies on diabetic patients have indicated that targeting albuminuria may improve survival (30, 31). However, data on such targeted therapy for albuminuria in heart failure patients is limited. In one of the included studies, Selvaraj et al. (18) demonstrated that the use of spironolactone in heart failure patients reduced albuminuria and improved the outcomes. Thus, further studies are warranted to elucidate the role of targeting albuminuria in heart failure patients to improve outcomes.

For decades, 24-h urine collection has been the gold standard to measure albuminuria. In recent times, dipstick urine analysis has gained popularity owing to its ease of use and rapid results (32). However, dipstick proteinuria is only sensitive to albumin and is a semi-quantitative measure rather than determining the exact values of proteinuria. Furthermore, it solely depends on the urinary protein concentration rather than the actual protein excretion rate (33). Despite its limitations, it has been a popular marker for assessing prognosis in large screening programs owing to its substantially low cost as compared to 24-h urine analysis (34). In a study on the general population, Konta et al. (35) have demonstrated the presence of microalbuminuria in 12, 65, and 92% of patients with dipstick proteinuria results of (–), (±), and (≥1+), respectively. As a supplementary analysis of our main results, we also assessed the prognostic value of this simple test in heart failure patients. Our results demonstrated a statistically significant 54% increased risk of all-cause mortality in heart failure patients with positive dipstick proteinuria. While 24-h urine albuminuria measurements shall remain the gold standard, dipstick proteinuria can be used as a rapid screening tool to predict adverse outcomes in such patients.

The results of our study should be interpreted with the following limitations. Firstly, our review could not include a large number of studies due to the paucity of such investigations in literature. The number of studies included in the meta-analysis of outcomes other than all-cause mortality was very low. Secondly, due to non-availability of data, we could not classify outcomes based on types of heart failure, i.e., with and without reduced ejection fraction. Thirdly, study patients were classified as positive for albuminuria or proteinuria based on single tests. Fluctuations during the course of the disease were not taken into account, and this may have caused a misrepresentation of some patients. The studies also differed in the timing of the albuminuria or dipstick proteinuria test relative to the identification of heart failure. At this point, it is not clear if any treatment strategy, which may have been initiated after the diagnosis, had an influence on the study results. Lastly, the number of confounding factors adjusted and the duration of follow-up varied across studies, and this may have influenced our results.

To conclude, our review indicates that both microalbuminuria and macroalbuminuria are predictors of mortality in patients with heart failure. Dipstick proteinuria may be used as a rapid screening test to predict mortality in these patients. Owing to the review limitations, the results of our study should be supplemented with future high-quality research. Studies should also be planned to analyze if albuminuria can be an independent target for therapy in heart failure patients to improve outcomes.

## Data Availability Statement

Publicly available datasets were analyzed in this study. This data can be found here: PubMed, Embase, ScienceDirect, CENTRAL, and Google Scholar databases.

## Author Contributions

WL and QL conceived, designed the study, and were involved in the writing of the manuscript. Q-yW, HY, and JY collected the data and performed the literature search. All the authors have read and approved the final manuscript.

## Conflict of Interest

The authors declare that the research was conducted in the absence of any commercial or financial relationships that could be construed as a potential conflict of interest.
